# Investigating the Psychological, Social, Cultural, and Religious Predictors of COVID-19 Vaccine Uptake Intention in Digital Age: A Media Dependency Theory Perspective

**DOI:** 10.3390/vaccines11081338

**Published:** 2023-08-07

**Authors:** Mengyao Ma, Syed Hassan Raza, Muhammad Yousaf, Umer Zaman, Qiang Jin

**Affiliations:** 1 School of Journalism and Communication, Hebei University, Baoding 071000, China; mamyao@stumail.hbu.edu.cn; 2 Institute of Media and Communication Studies, Bahauddin Zakariya University, Multan 66000, Pakistan; 3 Centre for Media and Communication Studies, University of Gujrat, Gujrat 50700, Pakistan; m.yousaf@uog.edu.pk; 4 Endicott College of International Studies, Woosong University, Daejeon 34606, Republic of Korea; 5 Intercultural Communication Research Center, Hebei University, Baoding 071000, China; jinqiang@hbu.cn

**Keywords:** vaccine hesitancy, COVID-19, social media, media dependency, religiosity, precautionary behavior, Pakistan

## Abstract

Media exposure to health communication contents related to the COVID-19 pandemic alone is inadequate to measure the influence of media on individuals in adopting precautionary behaviors against COVID-19, such as vaccine uptake. Certain individuals may pay attention to and be influenced by communication content. However, literature has suggested other instrumental determinants in developing and adopting health precautionary measures, such as culture or religion, especially regarding vaccination. In times of crisis, such as the COVID-19 pandemic, it is valuable to examine the interrelationships among psychological, sociocultural, and informational factors. This can provide valuable insights for policymakers in developing effective communication strategies. Drawing an analogy between the Media dependency theory (MDT) and the Theory of Planned Behavior (TPB) model, this study unravels the factors underpinning the COVID-19 vaccine uptake intention among Pakistanis. The study utilized a cross-sectional research design and employed a survey method to gather data from a sample of 993 participants. The findings obtained from the PLS-SEM analysis confirmed that individuals relied on both traditional and social media to cope with the COVID-19 pandemic. The findings show that individuals rely more on the informational content disseminated through conventional media channels. The findings also suggest that individuals from Asian countries, such as Pakistan, tend to be more inclined toward collectivism. The findings about the moderating role of religiosity suggest that religious beliefs significantly shape individuals’ reliance on traditional media. Hence, this study has uniquely contributed to public health and media management by providing a strategy for managers to address disseminating misinformation related to religion and its impact on vaccination-related health issues. The study has theoretically confirmed the principles of media dependency theory. As a result, it is recommended that various information sources be utilized to cultivate resilience among individuals to manage health crises effectively.

## 1. Introduction

The global pandemic of COVID-19 has contributed to serious threats to the lives of people across the globe, and the pandemic has already claimed hundreds of thousands of lives worldwide [1]. COVID-19 broke out in Wuhan, China, in late 2019, spread to more than 200 countries, and infected more than 9 million people globally by 19 June 2020 [2]. As the spread of COVID-19 was associated with human social life, people had certain responsibilities to lessen its spread. To this end, executing awareness activities, including media campaigns, became crucial for the masses [3]. Therefore, many nations started using traditional and social media to initiate precautionary behaviors (e.g., COVID-19 vaccine uptake intention). These efforts are contingent on the already known literature recommendations; for example, scholars [4] posited that media dependency increases in disasters, and people rely on the media to get desired information to guide their actions.

Global awareness campaigns were initiated in accordance with the guidelines mentioned above [5]. Numerous authorized entities attempted to enhance societal consciousness regarding precautionary measures and intervention strategies by disseminating routine updates on COVID-19 active cases through various media channels, including social media [6,7]. Despite the employment of diverse communication channels by various nations, it was noted that the impact of media campaigns varied among nations. As an illustration, certain nations, including Pakistan, reported persistent grievances from officials regarding the populace’s reluctance to embrace suitable precautionary measures, despite the government’s repeated endeavors to raise awareness on the matter [8]. Therefore, it is crucial to investigate why individuals exhibit varying degrees of adherence to media awareness campaigns promoting COVID-19 vaccine uptake, as this phenomenon is observed globally. Further research is needed to address this important issue.

Previous research [9,10] has extensively examined the influence of various antecedents, including media surveillance, subjective norms, and perceived severity, on developing COVID-19 vaccine uptake intention. However, contextual factors such as culture and religiosity, which may also affect health behaviors, have not been considered collectively in these studies. Moreover, certain scholarly investigations [11] have documented the effects of media campaigns launched during the COVID-19 pandemic. However, these studies have provided limited evidence regarding how individuals process such information and subsequently develop precautionary behaviors.

Numerous studies have emphasized the significance of media dependency in predicting preventive behaviors regarding risk information processing [12,13,14]. However, little research on health communication in the Asian context has indicated that exploring preventive health behaviors in Asian societies is multifaceted, hence the need to examine the impact of cultural context on individuals’ preventative behaviors [15,16]. Examining what prompts individuals to embrace preventive measures against COVID-19 in Asian contexts, including Pakistan, is essential owing to these regions’ diverse sociocultural beliefs and media landscapes.

Moreover, this study makes a scholarly contribution by employing a comparative approach regarding individuals’ dependency on diverse media formats, drawing upon established theories such as MDT and TPB. It addresses a gap in previous studies on COVID-19 by investigating a relatively unexplored phenomenon: how individuals respond to health-related information in their social and religious contexts. Therefore, this research examines how various health-related information disseminated on different media platforms influenced COVID-19 vaccine uptake intentions. As a result, the present study aims to elucidate factors that may prove advantageous in formulating a communication strategy, given its imperative role in addressing the urgent demands for public health safety, especially during a health crisis. The present research aims to provide policymakers with a comprehensive toolkit for implementing a suggestive communication strategy in response to health issues like the COVID-19 pandemic. This strategy involves the integration of traditional, social media, and interpersonal channels.

## 2. Literature Review

### 2.1. Theoretical Underpinning and Framework

Merely relying on media coverage is inadequate to fully gauge the impact of media on health-related preventive behaviors [12,17]. Media dependency pertains to the impact of media usage on the formation and enactment of human behavior. The media dependency theory (MDT hereinafter) provides an opportunity to tap the media’s influences at various levels of investigation [18]. Scholars have categorized media dependency theory into three levels (1) macro level, (2) micro level, and (3) individual level. The macro-level investigation is related to organizational dependency relationships between the media, social systems, and audiences. MDT suggests that individuals depend more on the media for information in uncertain situations such as crises or natural disasters [4].

Individuals’ dependency on media will have disproportional influence at the micro level. Individuals’ needs and fulfillment of the aims will depend on the information provided by social institutions or media organizations [19]. Hence, several factors, including the accessibility of alternate information resources and societal background dynamics, such as risk, can raise individuals’ dependence on the message [20]. Limited research [21] conducted at the individual level of media dependency considered greater influences on individuals’ attitudes or behaviors.

Literature shows that media dependency can knowingly forecast variations in media audiences’ behaviors [22]. For instance, it is revealed that factors such as news perspective and usage of slants predicted people’s uncertainty regarding the reasons and impacts of climate change [23]. Hence, it is plausible that the copious dissemination of information through conventional and digital media regarding COVID-19 could potentially exacerbate the risk of contagion by raising public awareness about the impact of COVID-19 on their health. However, other contributory factors, such as cultural perspectives and psychological mechanisms, are also crucial to understanding this phenomenon.

Extensive research that utilized various theoretical frameworks, including the Theory of Planned Behavior [24] (hereafter TPB), the Health Belief Model [25], and the Protective Motivation Theory [26], has been conducted. The Theory of Planned Behavior (TPB hereinafter) is considered a well-known theory in studying and understanding the psychological mechanisms related to the development of health behaviors, and the Theory of Planned Behavior (TPB hereinafter) is considered a well-known theory [27]. TPB has offered constructs such as attitudes, subjective norms, perceived behavioral control, and behavioral intention [28]. By doing so, TPB has underpinned the psychological and cultural factors that can predict behavioral intention. Behavioral intention is a substitute for measuring real behavior [29]. TPB has been widely used in explaining the numerous health-related diverse outcomes, such as hand hygiene [30], dairy product consumption [31], smoking [32], and physical activity [33]. Furthermore, many meta-analyses have also validated the efficacy of its predictors in determining health-related outcomes [28]. It is noted that TPB has validated its usefulness in forecasting health behaviors among other information processing and social cognitive theories [34]. The comparative analysis of health behavior theories in different behavioral domains has demonstrated the effectiveness of TPB in predicting a diverse range of health-related behaviors. This effectiveness is supported by several strengths, including the addition of constructs that assess cognitive mechanisms, normative aspects, and individual motivational factors [24].

The attitude construct in TPB is an individual’s extent of liking or disliking the behavior in question and directs the steady behavioral outcomes [29]. Hence, it can reflect contributory attributes (i.e., helpfulness) and pragmatic attributes (i.e., pleasurableness) of action [12]. For instance, this perceived usefulness motivates some actions (i.e., washing hands), while amusement motivates others (i.e., viewing music); however, various actions echo both cognitive and affective orientations. To this end, it would be imperative to integrate the notion of MDT with TPB to know how Pakistani respondents will react to COVID-19-related media campaigns.

The other important predictor of behavior in TPB is the subjective norm, which is the individual’s perception of a peer’s endorsement or discontentment with the behavior in question [28]. According to TPB, individuals frequently make societal comparisons of their actions with those of their referent group members; they are more indubitably pretentious in their perceptions of in-group than out-group behaviors [12]. This study also considered collectivism as the antecedent for determining the subjective norm, which aligns with the Asian cultural tendency. The learned schemas of the culture guide the norms, and these social norms have varying impacts on behavioral patterns [35]. Therefore, it is eloquent to study how such cultural tendencies guide subjective norms, which serve as a mediating mechanism to develop vaccination intention in the case of COVID-19-related precautionary measures [27]. The perception of control is one of the numerous factors associated with an individual’s engagement in healthy behaviors. Perception of control refers to a person’s subjective perspective or comprehension of their influence or authority over a given situation or outcome. The capacity to ascertain or exert control over something is called perceived behavioral control [36]. In the present context, the term ‘something’ refers either to the behavior in question or to the consequences resulting from the said behavior. Consider, for instance, the health-related practice of seeking medical attention for a comprehensive physical examination every year. In the context provided, perceived control encompasses an individual’s belief regarding their ability to exercise agency in deciding whether or not to seek medical attention, determining the timing of such visits, and even selecting a preferred healthcare provider.

Additionally, it can also encompass the belief that one can enhance or sustain their state of health by undergoing routine medical evaluations. In both scenarios, individuals who perceive higher control over a given situation, whether over their behavior or the resulting consequences, are more inclined to engage in the specific behavior. This assertion holds true when all other factors remain constant. In the context of COVID-19 vaccine uptake, PBC is more critical, as the behavior in question involves several aspects, such as side effects that can hinder their intention [24]. The PBC might not be effective in other contexts, such as making purchase decisions; however, in a particular scenario of the COVID-19 vaccine, where a plethora of misinformation is accessible to individuals, it becomes a crucial factor. The willingness to get a COVID-19 shot is not comparable to one’s ability to purchase a bicycle. Therefore, in the current study, we postulate that the perceived behavioral control of individuals would predict the COVID-19 vaccine uptake intention.

**Figure 1:**
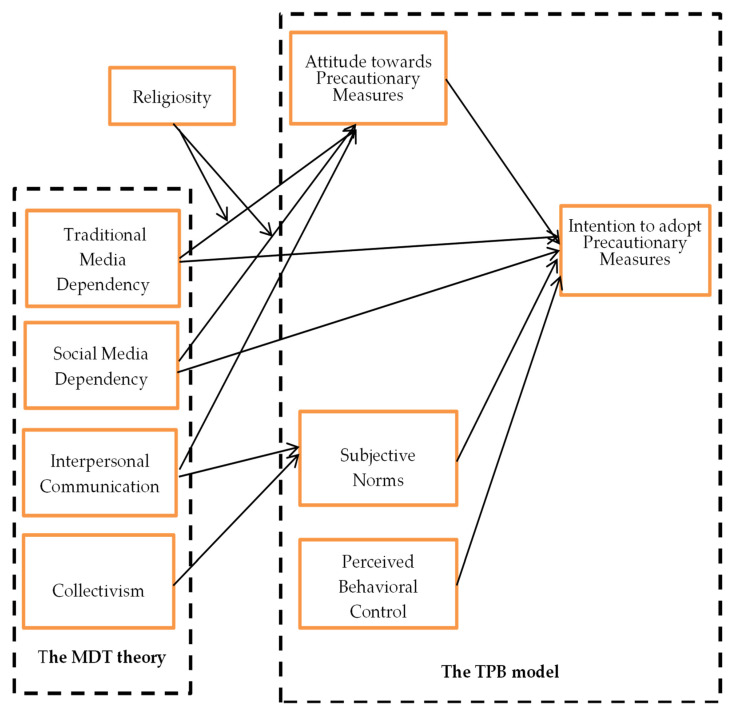
Conceptual Model.

In the given research context, the TPB emerges as a prominent theoretical framework for information processing [37]. The integration of TPB has encompassed various factors pertaining to communication, including media attention and social media platforms, to elucidate health behavior [37]. Moreover, the utilization of the TPB theoretical framework in this research incorporates context-specific variables such as religiosity that address the limitations observed in prior studies on the adoption of health behaviors. The TPB outlines the decision-making process at the individual level [38]. This study has incorporated the theoretical principles of the MDT, which are essential in understanding the adoption of health behaviors, such as the increased uncertainties surrounding COVID-19 vaccines. The MDT suggests that individuals turn to the media to fulfill their informational needs and subsequently influence their behavioral outcomes, particularly in uncertain situations. Thus, drawing an analogy between MDT [4] and TPB [27], we assume that people’s reliance on traditional and social media will improve the information during a crisis. It is noted that such dependency on media outlets may predict deviations in individuals’ attitudes regarding COVID-19 vaccine uptake intention [14,21]. However, considering the contextual factors, we assume that not only does this dependency determine individuals’ behaviors, but factors such as religious tendency can intensify or diminish this dependency. For example, some Pakistani religious clerics asked people not to follow the COVID-19-related instructions disseminated in the media, which altered the people’s attitude. Thus, we propose that it is crucial to consider factors such as religiosity or collectivism to underpin the development of precautionary behavior in Asian countries. In doing so, this study attempts to integrate MDT and TPB to explain certain levels of media dependence and cultural factors such as norms and religion. Therefore, we proposed the following conceptual model in Figure 1.

### 2.2. Hypotheses Development

#### 2.2.1. Traditional Media and Social Media Dependency

Media dependency refers to how individuals rely on the media for information regarding politics, education, culture, and entertainment. Media dependency has been widely investigated in the field of mass communication. MDT was conceptualized by Ball-Rokeach and DeFleur [4] to measure (micro) individual level, (meso) group or organizations dependency, and (macro) social systems. The macro level pertains to the interdependent relationships among audiences, mass media, and other social systems at a structural level. At the micro level, media dependency theory reveals that individuals depend on information gathering, processing, and disseminating for their daily activities [19]. The individual dependencies on media for the aforementioned purposes depend on several alternative sources of information [17]. And these individuals’ dependence on mass media as a source of information increases during crises, uncertainty, and societal disruptions. This dependency has significant consequences for understanding the role of media in modern complex-mediated societies [4,19]. It is assumed that merely relying on media coverage is insufficient for predicting the impact of media coverage on health-related issues such as COVID-19. In other words, media content consumption could not be perceived as a determinant factor in shaping the conduct of individuals.

Nonetheless, the scientific community paid little attention to individual-level media dependency effects. In this regard, Ref. [21] a survey conducted to document media dependency in the aftermath of the terrorist attacks on September 11 found that individual-level media dependency was a significant predictor of attitudinal and behavioral changes in the respondents. In the context of social media dependencies, Ref. [39] found that social networking sites’ “dependency had direct effects on individual users’ levels of engagement with interactive activities on SNSs and indirect effects on offline interpersonal storytelling” (p. 1458). Likewise, scholars [40] concluded that individuals’ dependence on YouTube provided them with a means to meet their emotional needs in times of crisis, such as the death of a public figure. Similarly, studies show that media messages enhance the willingness to accept vaccines [41,42,43]. Keeping this literature in view, we proposed the following hypotheses:

**H1.**  *Traditional media dependency positively relates to (a) attitude towards precautionary measures and (b) COVID-19 vaccine uptake intention.*

**H2.**  *Social media dependency positively relates to (a) attitude towards precautionary measures and (b) COVID-19 vaccine uptake intention.*

#### 2.2.2. Interpersonal Communication and COVID-19 Vaccine Uptake Intention

Interpersonal communication is the verbal and nonverbal transfer of information and messages between two or more people. It entails the exchange of ideas via the use of words, body language, facial expressions, and other types of communication. In many contexts, including interpersonal relationships, business, education, and healthcare, among others, effective interpersonal communication is crucial. It involves attentive listening, empathy, and the capacity to comprehend others’ needs and viewpoints and to respond effectively to them. Interpersonal communication can replace mass communication to convey information and influence people’s actions. A study found a link between risk perceptions and interpersonal discussion of health concerns. The study does not, however, give enough details on how this link came to be [44]. Interpersonal communication has been recognized in earlier research as the primary source of social norms and perceived efficacy, and it has also been shown to impact people’s attitudes and behaviors [45].

**H3.**  *Interpersonal communication is related to (a) attitude towards precautionary measures and (b) subjective norms.*

#### 2.2.3. Collectivism

Culture is the collective set of ideas, standards, and practices created by human societies to address social and environmental issues [46]. Societies must effectively manage substantial environmental risks like disease-causing viruses, and cultures have developed in response to these difficulties. The prevalence of infectious diseases in ecological areas has been associated with the development of a cultural syndrome known as collectivism [47]. The concept is rooted in the fundamental psychological differentiation between a self-perception that is interdependent with others and one that is independent and distinct from others [48]. The collectivism-individualism dichotomy is crucial for capturing cultural variance [49]. The link between pathogen prevalence and collectivism apparently arises due to some characteristics of a collectivist society serving as barriers to disseminating contagious illnesses. In particular, individuals who adhere to collectivist values tend to exhibit greater wariness when engaging with unfamiliar individuals, potentially mitigating the risk of encountering novel infections. They also place high importance on customs like food preparation, which might act as barriers to spreading COVID-19 [38].

Although it has been suggested that collectivist societies may be better able to control the dissemination of contagious illnesses, other aspects of collectivism may work against this goal. Individuals connect more frequently and closely in collectivist communities [48], which helps infectious diseases spread [50]. Collectivist societies have a propensity to marginalize the underprivileged (such as those from lower socioeconomic status) and are linked to ineffective social programs for disadvantaged groups [49]. These elements contribute to the spread of infectious diseases. Given these conflicting claims, it is unclear to what extent collectivism can successfully combat a severe epidemic like COVID-19.

On the other hand, an alternative body of literature posits an inverse association between collectivism and proactive health practices [46]. It is demonstrated that the sociability facet of extraversion, which pertains to the inclination towards social activities and a preference for socializing over solitude, is positively linked to the transmission of COVID-19 [51]. This sub-dimension is a characteristic of collectivism [48]. Given the increased likelihood of COVID-19 transmission in public settings, it is reasonable to suggest that the conduct of individuals who prioritize collective interests may be significantly impacted by their perceptions of societal norms surrounding preventative measures. The present argument posits that a collectivist mindset is more conducive to promoting preventive behaviors, provided societal norms widely accept such behaviors as appropriate. And we hypothesized that:

**H4.**  *Collectivism is positively related to subjective norms.*

#### 2.2.4. The Role of Attitude, Subjective Norms, and Behavioral Control

TPB is considered a framework for understanding the psychological foundations of intentional behavior in humans [27,38]. This hypothesizes three primary antecedents to the formation of intentional behavior. These include attitudes, subjective norms, and perceived behavioral control. Ajzen and Fishbein contend that behavioral intention is a reliable indicator of actual behavior [52]. Perceived behavioral control (PBC) pertains to an individual’s evaluation of their ability to execute a specific behavior of interest. The level of perceived behavioral control is subject to variation depending on the situation and corresponding actions, as it is contingent upon an individual’s perception of their ability to regulate their behavior in different contexts [27,33]. The TPB supplanted the Theory of Reasoned Action after adding the PBC [53]. The concept of perceived behavioral control is analogous to perceived self-efficacy, a central tenet of social cognitive theory [54]. Abundant research has demonstrated the generalizability of the TPB framework to a wide range of contexts [55,56].

Studies have demonstrated that individuals are more likely to develop a stronger sense of perceived control when confronted with a greater level of threat, such as the contagious COVID-19 [57]. For instance, an individual’s conviction that implementing precautionary measures such as vaccination can effectively mitigate potential risks contributes to developing their PBC. The TPB suggests a positive relationship between PBC and behavioral intention, indicating that individuals are more likely to feel confident in their ability to take action when they perceive a high level of control [38]. Hence, in situations characterized by high risks, such as COVID-19, it is anticipated that individuals will exhibit positive behavioral modifications, such as a heightened willingness to adhere to COVID-19 preventive measures, as a result of the activation of the danger control mechanism. Previous health behavior research elucidates the intricate relationship among various cognitive constructs, such as PBC, that govern preventive outcomes [24]. PBC is a significant predictor of the response’s characteristics, specifically the feasibility of implementing a preventive response such as COVID-19 vaccine uptake intention [38]. Previous research has indicated that individuals exhibit an augmented sense of PBC primarily due to adequate prompts for engaging in safety-related behaviors related to COVID-19 vaccine uptake [29]. The level of confidence in individuals’ ability to effectively plan, implement, and oversee their future circumstances was found to be necessitating, as exemplified by the perception that COVID-19 vaccine uptake is a sustainable solution; thus, it is hypothesized that:

**H5.**  *Perceived behavioral control is positively related to COVID-19 vaccine uptake intention.*

Subjective norms pertain to an individual’s perception regarding the consensus of societal approval or disapproval of a particular behavior. This pertains to an individual’s perceptions regarding their peers’ and significant others’ social norms and expectations concerning their engagement in a specific behavior. The norms could be categorized as descriptive and injunctive norms, which could manifest at both individual and communal levels. Individuals seek the degree of endorsement of a particular behavior within their peer groups and in broader society [58]. People socially compare their behaviors with those of their peer groups and are more vulnerable to the influence of in-groups than out-groups [59]. Previous research has demonstrated that descriptive and injunctive norms have differential effects on behavioral intentions [60,61]. Collectivistic societies differ in terms of assigning significance to subjective norms. Those who live in collectivistic societies give more importance to subjective norms, whereas those residing in individualistic societies tend to prioritize PBC in decision-making. We propose the following hypotheses:

**H6.**  *Subjective norms are positively related to COVID-19 vaccine uptake intention.*

In Fishbein and Ajzen’s conceptualization, attitudes refer to an individual’s favorable or unfavorable evaluation of a particular behavior object, which subsequently corresponds to individuals’ behavioral responses [62]. Attitudes may specify instrumental qualities, such as usefulness, and experiential qualities, such as pleasantness, of a particular behavior [63]. For instance, behaviors such as tooth brushing are utility oriented, while behaviors such as watching a movie are pleasure-seeking-oriented [64]. However, several behaviors reflect a combination of both utility and enjoyment orientations. The presence of a pessimistic outlook regarding vaccines and the reluctance of individuals to undergo the COVID-19 vaccination constitute a significant public health issue in managing the pandemic [65,66]. Attitude refers to a concise representation of the assessment of a psychological entity. For instance, individuals may have varying perceptions regarding the positive or negative aspects and the potential risks or safety associated with receiving a vaccine. According to the TPB, an individual’s behavioral intentions are determined by their attitude towards the behavior. Prior studies have demonstrated that individuals’ attitudes toward vaccination are reliable indicators of their intentions to get vaccinated [67,68]. In order to enhance vaccination intentions, it may be necessary to strengthen general attitudes toward vaccines [69]. Therefore, previous research has consistently confirmed the significant impact of attitude on vaccination intentions.

**H7.**  *Attitude towards precautionary measures mediates the relationship between (a) traditional media dependency, (b) social media dependency, and (c) Interpersonal communication with COVID-19 vaccine uptake intention.*

#### 2.2.5. The Moderating Role of Religiosity

Religious beliefs are also accorded substantial attention regarding COVID-19 precautionary behavior [70]. The impact of religious beliefs on attitudes toward vaccination can be discerned as individuals endeavor to harmonize scientific realities with their theological framework, leading to a heightened propensity for vaccine refusal [43,71]. The notion of religiosity pertains to the nature of an individual’s religious convictions and encounters and religion’s function within a given society. There is a significant correlation between religiosity and COVID-19 vaccine uptake intention. The concept pertains to the conviction that every event occurs solely at the discretion of the divine [72]. Hence, adherents are expected to conduct themselves in accordance with the precepts of God [73]. Literature indicates that scholars have directed their attention toward religiosity in the context of the COVID-19 pandemic [74]. Several studies revealed notable correlations between religiosity and various health behaviors [72,75,76,77]. The results of these studies suggest a positive correlation between higher levels of religiosity and an increase in health-protective behaviors. Other studies revealed that individuals who identify as religious exhibit a heightened level of concern regarding the pandemic compared to those who do not identify as religious [78]. Hence, enhancing one’s religious beliefs could potentially foster spiritual and psychological fortitude amidst the ongoing pandemic [79]. Several studies have observed that religious affiliation fosters effective coping mechanisms and confers psychological advantages, thereby mitigating the likelihood of developing depressive symptoms in the context of the pandemic [80,81]. These studies show insistent results; therefore, we propose that

**H8.**  *Religiosity moderates the relationship between (a) traditional media dependency and (b) social media dependency with an attitude towards precautionary measures.*

## 3. Materials and Methods

### 3.1. Design, Participants, and Procedure

In the new communication environment, behavioral intervention programs have been diversified, and the usage of multiple means of communication has made the promotional phenomenon more complex. The current study used a cross-sectional research design via-a-vis a survey method to examine media dependency patterns in diverse settings. To do so, generalized data will be collected from Pakistan using a multistage sampling technique. Before data collection, the sample size was determined using two established methodologies: (1) Morgan’s Table and (2) G-power analysis. Based on current statistical data, the estimated number of internet users in Pakistan is approximately 89.3 million. Based on Morgan’s estimation, a total of 384 participants were deemed suitable. Subsequently, a G-power analysis was performed to ascertain the necessary sample size for this investigation, taking into account the number of variables of interest. The results indicated that a sample size of 950 participants would be sufficient to generate predictions and inferences from the data, given the presence of five antecedents and one dependent variable. Although the sample size of 800 participants was sufficient for making predictions and drawing inferences, the study aimed to collect an additional 10% of data to address potential challenges related to data normalization in the context of structural equation modeling (SEM). In total, 993 responses were obtained, and a comprehensive analysis of the findings is presented in the subsequent section. The questionnaire and corresponding definitions of constructs were disseminated to six academic professionals for feedback and to ensure translational validity before the commencement of data collection. Slight modifications were implemented in accordance with the feedback provided by the individuals. Subsequently, a preliminary investigation was conducted, encompassing a sample of 50 students. The study’s findings indicate that the scales’ reliability was deemed satisfactory, with a value of less than 0.60. The ultimate survey comprised various items and constructed definitions. The subsequent section expounds on the specifics of the tools employed in this study.

In developing measuring instruments, the researchers collected and adapted available instruments. Furthermore, based on the nature of the study, the selected instruments were translated into the local language. A back-translation technique was applied, and translational validity was attained by using face validity ratings from six experts (academicians). Upon getting feedback from the experts, the suggested amendments were made. Before carrying out the main study, a pilot study using 30 participants was conducted. Pakistan is considered for this research as it is a religiously sensitive country. Data analysis was desired to reveal whether psychological (i.e., attitude) and contextual factors (i.e., religiosity) play any role in determining the impacts of media dependency. Finally, for analysis, PLS was used, as it is the right approach to deal with complex moderating-effect models.

This study utilized a cross-sectional field survey method to collect data from a sample of 993 adults in Pakistan. The data was gathered from April to August 2022, utilizing enumerators from a specialized data collection firm. During a training session, the lead researcher provided a briefing to six enumerators associated with the local data collection firm. The briefing covered the primary objectives of the research as well as the sampling and data collection techniques to be employed. Data collection for the primary survey was carried out using a multistage sampling technique, specifically employing random and convenient sampling methods. The study used two sampling techniques to select the appropriate respondents. In the first stage of the study, a random sampling technique was utilized to select ten administrative units, such as districts, from Pakistan. The lottery method was employed for this purpose. Subsequently, a compilation comprising the names of subordinate units, such as sub-districts, was generated for each district that had been chosen. During this phase, a total of ten smaller administrative units were chosen from the list mentioned above. The data collection was conducted using a convenient sampling method. During the purposive sampling phase, the enumerators visited designated areas and included participants who consented to participate in this study. Subsequently, the participants were requested to complete the questionnaire, and the data was entered into a digital format using tablets and a Google Form.

### 3.2. Instrumentation

#### 3.2.1. Attitude towards Precautionary Measures

We modified three questions from Ajzen’s research [82] to gauge respondents’ attitudes towards precautionary measures on a semantic scale that taking precautionary measures against COVID-19 is (1) worthwhile, (2) significant, and (3) pleasant, anchoring “1 = strongly disagree, 5 = strongly agree”.

#### 3.2.2. Subjective Norms

In order to measure subjective norms, we adjusted four questions from Park and Smith’s work [61]. Respondents were asked to rate how strongly they agreed or disagreed with the assertions that their family members, close friends, and the general public would approve of their precautionary measures against COVID-19 on a five-point Likert scale, anchoring “1 = strongly disagree, 5 = strongly agree”.

#### 3.2.3. Perceived Behavioral Control

PBC respondents gave their opinions on the following three statements adapted from literature [82], which read as follows: “It is possible for me to take COVID-19 vaccine shot”. “It is up to me whether I take COVID-19 vaccine shot…”, “I believe I have complete control over taking COVID-19 vaccine shot…”, on a five-point Likert scale anchoring “1 = strongly disagree, 5 = strongly agree”.

#### 3.2.4. COVID-19 Vaccine Uptake Intention

We modified three questions from Ajzen’s work [82] to gauge respondents’ COVID-19 vaccine uptake intention on a Likert scale anchoring “1 = strongly disagree, 5 = strongly agree”. The items read as follows: (1) “I intend to get the COVID-19 vaccine booster dose”. (2) I am willing to receive a COVID-19 vaccine booster dose” and (3) “I plan to receive a COVID-19 vaccine booster dose as the media is advocating it”.

#### 3.2.5. Media Dependency: Traditional Media and Social Media

We adapted three items from Loges’ research [83] to measure participants’ reliance on traditional media. The participants expressed their concurrence with the following four statements. Reading newspapers and watching TV “helps me find out about precautionary measures against COVID-19”, “helps me observe how others deal with COVID-19”, and “helps me figure out how I can protect myself from COVID-19”. The same statements were re-worded for participants who relied on social media. For instance, viewing social media “helps me find out about precautionary measures against COVID-19”. All items were measured on a five-point Likert scale, anchoring “1 = strongly disagree, 5 = strongly agree”.

#### 3.2.6. Interpersonal Communication

Three items were used to measure the participants’ reliance on interpersonal communication based on their discussions with friends, family, and colleagues about protection against COVID-19. I rely on discussions with (1) friends, (2) family, and (3) colleagues to learn about precautionary measures against COVID-19. All items were measured on a five-point Likert scale, anchoring “1 = Never, 5 = All the time”.

#### 3.2.7. Collectivism

Four items were adopted from the work of Oyserman et al. [84] to measure collectivism on a five-point Likert scale. The items read as follows: “(1) I make an effort to avoid disagreements with my group regarding precautionary measures against COVID-19, (2) Before deciding COVID-19 vaccine uptake, I always consult with others, (3) How I adopt precautionary measures against COVID-19 depends on who I am with, (4) I have respect for the authority figures with whom I interact, and my COVID-19 vaccine uptake decision would depend on his/her advice”.

#### 3.2.8. Religiosity

Three items were adopted from the literature [85,86] to measure religiosity on a five-point Likert scale. The items read as follows “(1) What religion offers me most is comfort in times of trouble like COVID-19, (2) Although I am religious, but I don’t let it affect my COVID-19 vaccine uptake decision, and (3) although I am religious, but I don’t let it affect my precautionary measure against COVID-19”. Items two and three were reversed.

## 4. Results

### 4.1. Demographic and Descriptive Analysis

The outcomes of the demographic analysis are shown in Table 1. The outcomes suggest diverse information concerning factors such as income, gender, educational achievement, and age.

Subsequently, the descriptive analysis was conducted using SPSS 24.0. This entailed the assessment of normality checks, outliers, variable correlation, and multicollinearity. The analysis of normality, both visually and statistically, indicated that there were initial issues pertaining to the normality of the data. The process of identifying and removing outliers was carried out through the application of outlier evaluations. Following the removal of 59 cases, the data was found to conform to normality assumptions based on the threshold values of skewness and kurtosis (±2.58) when adjusted by the standard error reported, in accordance with the recommendation of [87].

The Variance Inflation Factor (VIF) test was also performed to assess multicollinearity. The study observed that each item’s Variance Inflation Factor (VIF) values were below the threshold of 10. Consequently, in light of the absence of any multicollinearity concern in this investigation, the analysis progressed toward the Structural Equation Modeling (SEM) technique. Furthermore, the presence of method biases was detected by conducting Herman’s test via exploratory factor analysis (EFA) on SPSS. In order to achieve this, all the constituent items of each variable, namely AT, CVI, IPC, RL, SM, SN, TM, BC, and COL, were loaded onto a unified factor. The exploratory factor analysis (EFA) findings indicated a variance of merely 37.4%. Furthermore, no concerns were identified with respect to common method biases, thereby enabling the study to progress to the structural equation modeling (SEM) phase.

### 4.2. Confirmatory Factor Analysis

This study employed partial least squares (hereafter PLS) SEM. It encompasses a range of multivariate techniques that are used primarily for confirmatory purposes. In addition, PLS-SEM offers enhanced predictive precision and a substantially reduced likelihood of spurious correlations. Hence, variance-based SEM, such as PLS, is widely acknowledged as more advantageous than regression analysis. This is primarily attributed to its capability to illustrate the influence of direct and indirect independent variables [87]. In order to evaluate the measurement model’s fit, convergent and discriminant validity, and potential for multidimensionality, the current study used a PLS-based SEM. The items were loaded into the parent constructs of AT, CVI, IPC, RL, SM, SN, TM, BC, and COL for confirmatory factor analysis (CFA) using PLS 3.0. The Confirmatory Factor Analysis (CFA) findings suggest empirical differentiation between AT, CVI, IPC, RL, SM, SN, TM, BC, and COL (refer to Table 2).

**Figure 2:**
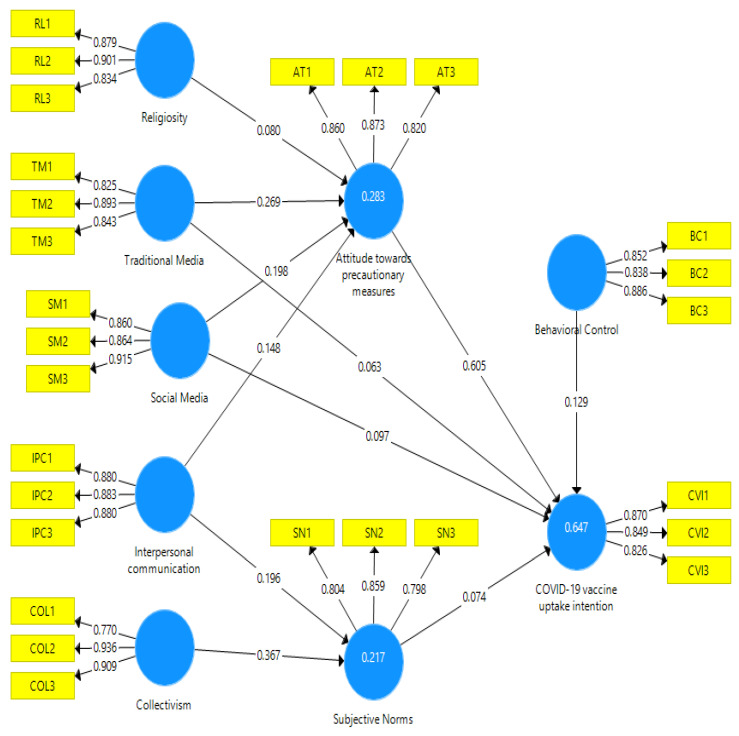
Measurement model.

Upon analysis, it was found that the model demonstrated a satisfactory fit after removing one item (SN4). Table 2 and Figure 2 present the reported item loadings.

Upon achieving the desired level of model fitness, the research proceeded to estimate reliability. The findings of the analysis indicate that the constructs of AT, CVI, IPC, RL, SM, SN, TM, BC, and COL exhibit satisfactory reliability estimates across multiple indices, including Dijkstra-Henseler’s rho (ρA), Jöreskog’s rho (ρc), and Cronbach’s alpha (α), as presented in Table 3. Various reliability indices, as introduced in PLS.3, were employed to perform cross-validation of the reliability estimations.

The study assessed the convergent and discriminant validities by utilizing the Fornell-Larcker criterion method. Convergent validity is established when the items within a specific measure exhibit convergence by effectively representing the latent construct being measured. The Average Variance Extracted (hereafter AVE) is computed by taking the average of the squared loadings of each indicator linked to a particular construct. Convergent validity is considered statistically established when AVE exceeds a threshold of 0.50. At the same time, discriminant validity is a crucial aspect of research methodology that aims to determine the distinctiveness of the constructs under investigation. The Fornell and Larcker Criterion posits that Discriminant validity can be deemed established if the square of the correlation between two constructs is less than the AVE for each construct. The fundamental basis of the AVE for a specific construct exhibited a higher magnitude than its correlation with all other constructs. These findings indicate that the constructs examined in the study possess distinct identities and exhibited moderate correlation with other constructs under investigation. The Confirmatory Factor Analysis (CFA) findings indicated that the Composite Reliability (CR) of all variables was acceptable, as presented in the Table. In addition, the study computed the Average Variance Extracted (AVE) and observed that the intercorrelations among AT, CVI, IPC, RL, SM, SN, TM, BC, and COL were below the square root of their respective AVE values (see Table 4). Subsequently, the research progressed to the application of inferential statistics.

### 4.3. Hypothesis Testing

This research posited numerous hypotheses to map the psychological and sociocultural influences on the precautionary measures regarding COVID-19. The hypotheses delineating the direct impact of traditional media (H1a), social media (H2a), and interpersonal communication (H3a) on attitude were supported. The results of the SEM using PLS revealed that there is a positive influence of traditional media (β = 0.258), social media (β = 0.198), and interpersonal communication (β = 0.174) on attitude. Furthermore, the results demonstrated that traditional media (β = 0.063) and social media (β = 0.097) significantly predict COVID-19 vaccine uptake intention. Therefore, H1b and H2b were also supported. Likewise, results suggested that interpersonal communication (β = 0.196) and collectivism (β = 0.367) positively predict subjective norms, as posited in H3b and H4. Finally, results verified that behavioral control (β = 0.129) and subjective norms (β = 0.074) predict COVID-19 vaccine uptake intention significantly. Thus, H5 and H6 were also supported.

Later, the mediation analysis revealed that attitude partially mediates (β = 0.163) the relationship between traditional media and COVID-19 vaccine uptake intention. Similarly, partial mediation of attitude (β = 0.120) was also found in the relationship between social media and COVID-19 vaccine uptake intention. Thus, H7a and H7b were also supported by the evidence presented in Figure 2 and Table 5.

**Figure 3:**
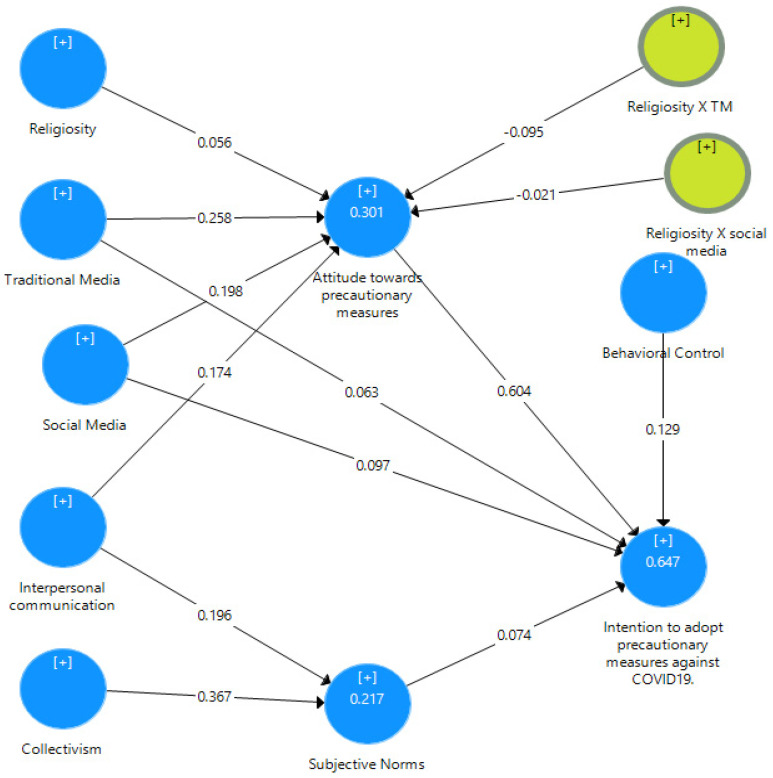
Structural model.

The present study posited the moderating influence of religiosity in determining the influence of the (H8a) traditional and (H8b) social media on attitudes. To determine the moderating influence specifically, the model included the two interaction terms (e.g., religiosity X traditional and religiosity X social media). Surprisingly, the analysis findings indicate no significant moderation influence of religiosity on the relationship between (H8a) traditional media and attitude; thereby, H8a was not supported. The findings of the analysis specify that there is a significant moderation effect of religiosity on the relationship between (H8b) social media and attitude (see Table 6 and Figure 3). The findings indicate that social media’s impact on attitude is a function of religiosity. These results were statistically significant and supported hypothesis H8b. Overall, the model extracted 64.7% variance with a reasonable effect size, as presented in Table 6.

## 5. Discussion

This study used a cross-sectional research design vis à vis survey method to examine the influence of traditional media dependency, social media dependency, interpersonal communication, and collectivism on COVID-19 vaccine uptake intention. The study also investigated the mediated role of attitude towards precautionary measures, subjective norms, perceived behavioral control, and the moderating role of religiosity. Overall, the study proposed thirteen hypotheses. Of the 13 hypotheses, nine explored the direct impact, two the mediating role, and two the moderating role. The nine direct hypotheses were supported. Likewise, three mediating hypotheses were also supported. Of the two moderating hypotheses, one was supported and the other was rejected. The findings of hypotheses H1a and H1b revealed that traditional media dependency influenced attitudes towards precautionary measures and COVID-19 vaccine uptake intentions.

Likewise, social media dependency impacts attitudes toward COVID-19 vaccine uptake intention, supporting hypotheses H2a and H2b. Put differently, the higher the media dependency on both traditional and social media, the higher the impacted attitude toward precautionary measures and COVID-19 vaccine uptake intention. These findings support the results of previous literature suggesting that higher media dependency during a crisis increases information seeking. This corresponds to a higher attitude toward adopting precautionary measures and boosts public COVID-19 vaccine uptake intentions [4,12]. Similarly, during uncertain times such as the COVID-19 crisis, individuals’ interpersonal communication with family, friends, and colleagues was also enhanced many-fold. This increased interpersonal communication corresponds to an increased awareness of COVID-19. Consequently, these encounters impact their attitude toward precautionary measures and their intention to adopt them against COVID-19 supporting hypotheses H3a and H3b. This finding supports the previous literature [17].

Those who live in collectivist cultures adhere more to subjective norms. People from collectivist cultures stay in collectivist communities. The adherence to collectivism apparently arises due to some characteristics of a collectivist society, which serve as a barrier to spreading infectious illnesses. In particular, collectivists are more cautious when interacting with strangers. This results in few exposures to novel infectious diseases. They also place high importance on cleanliness, such as in customs like food preparation, which might act as barriers to spreading COVID-19. These findings align with the previous outcomes [46]. H5 posited that perceived behavioral control positively relates to COVID-19 vaccine uptake intention. The findings support the hypothesis. Perceived behavioral control (PBC) is related to an individual’s subjective assessment of their behavioral response, and it varies across situations and actions based on contexts [27]. It corresponds to precautionary measures against COVID-19 (i.e., vaccine uptake).

The present study provides empirical evidence that supports the theoretical constructs associated with the intentions of vaccinated individuals to seek a COVID-19 booster vaccine. The factors pertaining to attitudes and subjective norms exhibited the most substantial effect sizes, suggesting that individuals’ perceptions regarding the value and usefulness of the booster vaccine and the opinions of influential individuals significantly influenced their behavior. The findings align with previous studies that examined the acceptance of an initial COVID-19 vaccine. Therefore, they substantiate that individuals exhibit utilitarian behavior and prioritize the viewpoints of individuals they deem significant, such as their immediate family and close acquaintances [88,89]. Despite being less pronounced, the impact of risk perceptions and perceptions of control was equally significant. Previous research has primarily emphasized vaccine risk perceptions and self-efficacy, with relatively less attention given to the primary vaccine [90]. There exists a possibility that individuals who have undergone vaccination procedures may have previously formulated and executed intentions to partake in identical endeavors. Consequently, due to their prior exposure and the accessibility of immunizations, individuals’ perceptions regarding the inherent risks associated with vaccination may have diminished, thereby bolstering their willingness to undergo vaccination once more. Consequently, this could potentially diminish the extent to which individuals rely on said information to decide their future choices regarding vaccination.

H6 posited that there exists a positive correlation between subjective norms and the COVID-19 vaccine uptake intention. Subjective norms refer to how individuals and communities approve or disapprove of a particular behavior. This involves individuals seeking support for a specific behavior within their social circles and in wider society. The results of our study provide evidence in favor of the hypothesis that subjective norms are linked to adopting precautionary measures against COVID-19, as previously suggested by studies [58,59]. Furthermore, it is supported that the correlation between (a) reliance on traditional media and (b) reliance on social media is mediated by the attitude towards precautionary measures. The results indicate that individuals with a more positive attitude towards precautionary measures are more inclined to adopt preventive measures against COVID-19, thereby supporting hypotheses H7a and H7b. The results above corroborate existing literature affirming that media communications impact individuals’ inclination to receive COVID-19 and Polio vaccinations [42,43,91].

Furthermore, a notable association between religiosity and adopting precautionary measures is observed. Individuals who strongly adhere to religious teachings tend to exhibit a greater inclination toward believing that all events are subject to the will of a higher power. The results of our study indicate that individuals who self-identify as religious and rely on conventional media as their primary source of COVID-19 information exhibit a lower propensity to adopt precautionary measures compared to those who are religious and rely on social media for COVID-19 information. These findings support hypotheses H8a and H8b, as previously proposed in the literature [78,79,92].

### 5.1. Theoretical Contributions

Recognizing the actions identified to lessen infection risk is a strategic aim for behavioral researchers during the COVID-19 pandemic. It can offer a substantiated base of possibly manipulative reasons as objectives for interventions to promote health-related behavior and prevent COVID-19 pandemics. During health-related crises like the COVID-19 pandemic, identifying the predictors of health behavior becomes imperative. This study investigates the probable psychological and cultural factors involved in developing COVID-19 vaccine uptake intentions. In doing so, the study has proposed a conceptual model that can effectively measure the tendency to media dependency among individuals. To this point, it can provide evidence-based facts on how behavioral intrusions could be attained during crises.

This study aims to evaluate the factors associated with promoting health-related behavior. This study proposed applying assumptions based on MDT and TPB to predict and explain health behavior. These assumptions help to predict the association of critical psychological and cultural factors related to the development of precautionary measures against COVID-19. Mapping and testing such processes allows this study to provide insight into many spheres. For instance, the evidence of informational dependency highlights the stipulated patterns and effectiveness of different modes of communication (traditional vs. social media).

On the other hand, the tradeoff between religiosity and culture in developing optimally operative interventions or not in promoting health precautionary behavior. This research’s theoretical contribution is associating the evidence of the contextual factors’ importance with the psychological process. Simultaneous inspection of the proposed model also gives insight into health behavior among individuals in an Asian nation. This research explored the moderating influence of religiosity, which has been widely observed in public discussions surrounding the COVID-19 pandemic. For example, an individual without specialized knowledge or expertise may justify their decision to avoid vaccination by attributing the outcome of their life to divine intervention. However, obtaining additional empirical evidence regarding one’s dependency on the media is necessary. This research provides additional support to previous findings within the framework of a predominantly Muslim country, demonstrating that media interventions can effectively mitigate the adverse impact of fatalistic beliefs on health-related outcomes.

### 5.2. Managerial Implications

This study has several managerial implications for communication and social marketing practitioners and governments in designing and updating their communication and health behavior intervention programs in the future in times of crisis. The results of this investigation offer enhanced and in-depth comprehension for professionals in the field of public health to address the issue of reluctance toward COVID-19 vaccination. This study provides practical insights for relevant authorities regarding the strategic utilization of communication resources to mitigate obstacles, such as religious beliefs and societal misinformation, that impede the acceptance of COVID-19 vaccines on social media platforms. The scholarly literature suggests that in situations where obstacles give rise to doubts about vaccines, increased media attention could expose media consumers to some information to counter misinformation, disinformation, or fake news. Well-planned media content may increase public knowledge about the advantages of vaccines and correspond to an increased willingness to take vaccines. In this regard, media practitioners could utilize a media dependency framework during times of crisis, such as COVID-19, to better serve the public for the common good.

On the other hand, vaccine hesitancy towards COVID-19 is regarded as an obstacle that diminishes public acceptance. Consequently, this research offers a significant strategic communication instrument for generating consciousness regarding the perceived risk of COVID-19 vaccination, enhancing the stakeholders’ perception and protective attitude towards COVID-19. This can be achieved by employing strategic communication messages to augment the acceptance of the COVID-19 vaccination. Finally, this study will also promote collaboration among health and media experts to launch, collaborate on, and execute future promotional programs together.

### 5.3. Limitations and Future Research

The present study’s results contribute to the existing literature by examining the acceptance of COVID-19 vaccines. However, the present investigation is subject to various constraints. The study employed a cross-sectional design, which captures a single area of the phenomenon being studied rather than providing a dynamic representation of the phenomenon through experimental designs. Henceforth, future research endeavors may employ longitudinal or experimental methodologies to monitor the evolving behavior concerning COVID-19 vaccine reluctance or to ascertain the factors contributing to the decrease in vaccine adoption. Furthermore, this study only measures exposure to media, and it is based on a narrow set of general traditional media and social media measures. Therefore, this study does not measure any media content or media perceptions. As such, its ability to make statements about media content and campaigns is quite limited. However, its contribution is focused on the intersection of religious culture and media culture as they influence individuals’ attitudes and intentions to adopt health behaviors in the context of precautionary measures for COVID-19 boosters. Thereby, it is recommended that future research incorporate the influence of news frames, documentaries, and other related factors. Besides, the study was conducted solely in Pakistan. Thus, the generalizability of its findings to other countries and contexts should be approached with caution.

## Figures and Tables

**Table 1:** Demographic Attributes of Respondents.

Demographic Attributes		Frequency	Percentage
Gender	Male	554	55.8%
	Female	439	44.2%
Marital Status	Single	451	45.4%
	Married	523	52.7%
	Divorced	19	1.9%
Educational status	High school or less	418	42.1%
	College Education	254	25.6%
	University education	321	32.3%

**Table 2:** Item standardized weights.

Items	AT	BC	COL	CVI	IPC	RL	SM	SN	TM
AT1	0.860								
AT2	0.873								
AT3	0.820								
BC1		0.852							
BC2		0.838							
BC3		0.886							
COL1			0.770						
COL2			0.936						
COL3			0.909						
IPC1					0.880				
IPC2					0.883				
IPC3					0.880				
CVI1				0.870					
CVI2				0.849					
CVI3				0.826					
RL1						0.879			
RL2						0.901			
RL3						0.834			
SM1							0.860		
SM2							0.864		
SM3							0.915		
SN1								0.804	
SN2								0.859	
SN3								0.798	
TM1									0.825
TM2									0.893
TM3									0.843

AT = Attitude towards precautionary measures, CVI = COVID-19 vaccine uptake intention, IPC = Interpersonal Communication, RL = Religiosity, SM = Social Media, SN = Subjective Norms, TM = Traditional Media, BC = Behavioral Control and COL = Collectivism.

**Table 3:** Reliability and Convergent Validity Statistics.

	Cronbach’s Alpha	rho_A	Composite Reliability	AVE
AT	0.811	0.817	0.888	0.725
BC	0.822	0.826	0.894	0.738
COL	0.842	0.845	0.906	0.765
CVI	0.806	0.813	0.885	0.720
IPC	0.856	0.858	0.912	0.776
RL	0.842	0.856	0.905	0.760
SM	0.856	0.885	0.911	0.774
SN	0.758	0.767	0.861	0.674
TM	0.814	0.816	0.890	0.730

AT = Attitude towards precautionary measures, CVI = COVID-19 vaccine uptake intention, IPC = Interpersonal communication, RL = Religiosity, SM = Social Media, SN = Subjective Norms, TM = Traditional Media, BC = Behavioral Control and COL = Collectivism, and AVE = Average Variance Extracted.

**Table 4:** Discriminant Validity.

Variables	AT	BC	COL	CVI	IPC	RL	S M	SN	TM
AT	0.851								
BC	0.569	0.859							
COL	0.328	0.381	0.875						
CVI	0.776	0.545	0.334	0.849					
IPC	0.324	0.392	0.308	0.369	0.881				
RL	0.357	0.240	0.438	0.377	0.293	0.872			
SM	0.415	0.279	0.466	0.456	0.268	0.527	0.880		
SN	0.397	0.307	0.427	0.451	0.309	0.560	0.558	0.821	
TM	0.462	0.361	0.465	0.488	0.371	0.481	0.500	0.705	0.854

AT = Attitude towards precautionary measures, CVI = COVID-19 vaccine uptake intention, IPC = Interpersonal communication, RL = Religiosity, SM = Social Media, SN = Subjective Norms, TM = Traditional Media, BC = Behavioral Control, and COL = Collectivism.

**Table 5:** Standardized Regression Weights.

Paths	Β	T Statistics	*p* Values	Hypotheses
Traditional Media → Attitude	0.258	4.77	0.000	H1 (a) Accepted
Traditional Media → CVI	0.063	1.39	0.003	H1 (b) Accepted
Social Media → Attitude	0.198	3.94	0.000	H2 (a) Accepted
Social Media → CVI	0.097	2.89	0.004	H2 (b) Accepted
Interpersonal communication → Attitude	0.174	2.61	0.009	H3 (a) Accepted
Interpersonal communication → SN	0.196	4.30	0.000	H3 (b) Accepted
Collectivism → SN	0.367	7.40	0.000	H4 Accepted
Behavioral Control → CVI	0.129	3.58	0.000	H5 Accepted
Subjective Norms → CVI.	0.074	2.78	0.047	H6 Accepted
TM → Attitude → CVI (Mediation)	0.163	4.85	0.000	H7 (a) Accepted
SM → Attitude → CVI (Mediation)	0.120	3.71	0.000	H7 (b) Accepted
(Religiosity X TM) → AT (Moderation)	−0.021	3.12	0.75	H8 (a) Rejected
(Religiosity X social media) → AT(Moderation)	−0.095	4.87	0.03	H8 (b) Accepted

AT = Attitude towards precautionary measures, CVI = COVID-19 vaccine uptake intention, IPC = Interpersonal communication, TM = Traditional Media, SM = Social Media, SN = Subjective Norms, COL = Collectivism, and β = Standardized Regression.

**Table 6:** Effect Size.

f Square	AT	CVI	SN
AT		0.594	
Behavioral Control		0.131	
Collectivism			0.316
Interpersonal communication	0.135		0.244
Religiosity	0.103		
Religiosity X TM	0.115		
Religiosity X social media	0.003		
Social Media	0.263	0.121	
Subjective Norms		0.107	
Traditional Media	0.327	0.095	

## Data Availability

The data supporting this study’s findings are available from the corresponding author upon reasonable request due to ethical and privacy restrictions.
